# Association of Acute Anti-inflammatory Treatment With Medium-term Outcomes for Coronary Artery Aneurysms in Kawasaki Disease

**DOI:** 10.1016/j.cjcpc.2022.05.007

**Published:** 2022-06-17

**Authors:** Kevin G. Friedman, Brian W. McCrindle, Kyle Runeckles, Nagib Dahdah, Ashraf S. Harahsheh, Michael Khoury, Sean Lang, Cedric Manlhiot, Adriana H. Tremoulet, Geetha Raghuveer, Elif Seda Selamet Tierney, Pei-Ni Jone, Jennifer S. Li, Jacqueline R. Szmuszkovicz, Kambiz Norozi, Supriya S. Jain, Angela T. Yetman, Jane W. Newburger, International Kawasaki Disease Registry, Carolyn A. Altman, Brett R. Anderson, Mikayla Beckley, Elizabeth Braunlin, Jane C. Burns, Michael R. Carr, Nadine F. Choueiter, Jessica H. Colyer, Frederic Dallaire, Sarah D. De Ferranti, Laurent Desjardins, Matthew D. Elias, Anne Ferris, Michael Gewitz, Therese M. Giglia, Steven C. Greenway, Kevin C. Harris, Kevin D. Hill, Michelle Hite, Thomas R. Kimball, Shelby Kutty, Lillian Lai, Simon Lee, Ming-Tai Lin, Tisiana Low, Andrew S. Mackie, Wadi Mawad, Kimberly E. McHugh, Tapas Mondal, Kimberly Myers, Michael A. Portman, Claudia Renaud, Rosie Scuccimarri, S. Kristen Sexson Tejitel, Karen M. Texter, Deepika Thacker, Sharon Wagner-Lees, Kenny Wong, Mei-Hwan Wu, Varsha Zadokar

**Affiliations:** a Boston Children’s Hospital, Harvard Medical School, Boston, Massachusetts, USA; b Division of Cardiology, Department of Pediatrics, The Hospital for Sick Children, University of Toronto, Toronto, Ontario, Canada; c Division of Pediatric Cardiology, Centre Hospitalier Universitaire Ste-Justine, University of Montreal, Montreal, Québec, Canada; d Division of Cardiology, Department of Pediatrics, Children’s National Hospital, the George Washington University School of Medicine & Health Sciences, Washington, District of Columbia, USA; e Department of Pediatrics, Stollery Children’s Hospital, Edmonton, Alberta, Canada; f Heart Institute, Cincinnati Children’s Hospital Medical Center, Cincinnati, Ohio, USA; g Department of Pediatrics, Rady Children’s Hospital-San Diego, University of California San Diego, San Diego, California, USA; h Department of Pediatrics, Children’s Mercy Hospital, Kansas City, Missouri, USA; i Department of Pediatrics, Division of Pediatric Cardiology, School of Medicine, Stanford University, Palo Alto, California, USA; j Pediatric Cardiology, Children’s Hospital Colorado, University of Colorado School of Medicine, Aurora, Colorado, USA; k Division of Pediatric Cardiology, Department of Pediatrics, Duke University Medical Center, Durham, North Carolina, USA; l Department of Pediatrics, Children’s Hospital of Los Angeles, Los Angeles, California, USA; m Division of Cardiology, Department of Pediatrics, University of Western Ontario, London, Ontario, Canada; n Maria Fareri Children’s Hospital at Westchester Medical Center Health, New York Medical College, Valhalla, New York, USA; o Department of Pediatrics, Children’s Hospital & Medical Center of Omaha, Omaha, Nebraska, USA

## Abstract

**Background:**

The impact of adjunctive anti-inflammatory treatment on outcomes for patients with Kawasaki disease (KD) and coronary artery aneurysms (CAAs) is unknown.

**Methods:**

Using data from the International KD Registry in patients with ≥ medium CAA we evaluate associations of treatment with outcomes and major adverse cardiac events (MACE).

**Results:**

Medium or large CAA was present in 527 (32%) patients. All were treated with intravenous immunoglobulin (IVIG), 70% were male, and the median age was 1.3 years (interquartile range: 0.4-4.0 years). The most common acute therapies included single IVIG alone in 243 (46%), multiple IVIG in 100 (19%), multiple IVIG + corticosteroids in 75 (14%), and multiple IVIG + infliximab + corticosteroids in 44 (8%) patients. Patients who received therapy beyond single IVIG had a larger CA *z*-score at baseline (*P* < 0.001) and a higher rate of bilateral CAA (*P* < 0.001). Compared with IVIG alone, early adjunctive treatments (within 3 days of initial IVIG) were not associated with time to CAA regression or MACE, whereas later adjunctive therapy was associated with MACE and longer time to CAA regression. Patients receiving IVIG plus steroids vs IVIG alone had a trend towards shorter time to CAA regression and lower risk of MACE (*P* = 0.07). A larger CAA *z*-score at baseline was the strongest predictor of an increase in the CAA *z*-score over follow-up, lower likelihood of CAA regression, and higher risk of MACE.

**Conclusions:**

Persistence of CAA and MACE are more strongly associated with baseline severity CAA than with acute adjuvant anti-inflammatory therapy. Patients who received late adjunctive therapy are at higher risk for worse outcomes.

## Résumé

**Contexte:**

L’incidence d’un traitement anti-inflammatoire d’appoint chez les patients atteints de la maladie de Kawasaki (MK) compliquée d’anévrismes coronariens est inconnue.

**Méthodologie:**

À partir de données provenant du registre international de la maladie de Kawasaki portant sur les patients ayant subi des anévrismes coronariens modérés ou importants, nous avons évalué l’incidence des différents traitements sur les résultats cliniques et les événements cardiovasculaires indésirables majeurs (ECIM).

**Résultats:**

Des anévrismes coronariens modérés ou importants ont été relevés chez 527 patients (32 %). Tous les patients recevaient des immunoglobulines administrées par voie intraveineuse (IgIV); 70 % d’entre eux étaient de sexe masculin, et leur âge médian était de 1,3 an (écart interquartile : de 0,4 an à 4,0 ans). Les traitements d’urgence les plus fréquents comprenaient un seul traitement par IgIV chez 243 patients (46 %), plusieurs traitements par IgIV chez 100 patients (19 %), une association de plusieurs traitements IgIV et de corticostéroïdes chez 75 patients (14 %) et une association de plusieurs traitements IgIV, de corticostéroïdes et d’infliximab chez 44 patients (8 %). Les patients ayant reçu un traitement autre qu’un seul traitement IgIV présentaient des scores *z* initiaux plus élevés pour le diamètre des artères coronaires (*P* < 0,001) et un taux plus élevé d’anévrismes coronariens bilatéraux (*P* < 0,001). En comparaison d’un traitement par IgIV seulement, les traitements d’appoint précoces (administrés dans les trois jours suivant le début du traitement par IgIV) n’ont pas eu d’incidence sur la durée avant la régression des anévrismes coronariens ni sur la survenue d’ECIM, alors que les traitements d’appoint plus tardifs ont été associés à un risque plus élevé d’ECIM et à une régression plus tardive des anévrismes coronariens. Les patients ayant reçu une association d’IgIV et de corticostéroïdes avaient tendance à présenter une régression plus rapide des anévrismes coronariens et un plus faible risque d’ECIM que ceux recevant uniquement un traitement par IgIV (*P* = 0,07). Un score *z* initial plus élevé pour un anévrisme coronarien était le facteur prédictif le plus puissant d’une augmentation du score *z* pendant la période de suivi, d’une probabilité plus faible de régression de l’anévrisme et d’un risque plus élevé d’ECIM.

**Conclusions:**

La gravité initiale de l’anévrisme coronarien est plus fortement associée à la persistance de l’anévrisme et à la survenue d’ECIM que le recours à un traitement anti-inflammatoire d’urgence en appoint. Les patients recevant un traitement d’appoint tardif étaient par ailleurs plus susceptibles de présenter des résultats défavorables.

Kawasaki disease (KD) is an acute vasculitis that preferentially affects medium-sized, muscular, extraparenchymal arteries, particularly the coronary arteries (CA).^1^^,^^2^ Although first described in Japan in 1967, KD is now recognized worldwide in patients of every racial and ethnic group.^1^ The incidence of KD is highest in individuals of Japanese ancestry and occurred in 359 per 1000,000 children aged 0-4 years in the 2018 Nationwide KD survey in Japan.^3^ CA involvement can range from transient, mild dilatation to giant coronary artery aneurysm (CAA). Patients with giant CAAs are at risk for major adverse cardiac events (MACE) including stenosis, thrombosis, myocardial infarction, and death.^1^^,^^4^^,^^5^ Intravenous immunoglobulin (IVIG) is well established as first-line therapy and significantly reduces the incidence of CAA.^6^ Despite appropriate IVIG therapy, approximately 5% of patients still develop CAA based on Japanese Ministry of Health criteria and up to 25% based on American Heart Association (AHA) criteria.^1^^,^^7^^,^^8^ For high-risk patients, several adjunctive therapies to standard IVIG have been evaluated, including corticosteroids, tumour necrosis factor α inhibitors (infliximab, etanercept), and the calcineurin inhibitor cyclosporine.^9^, ^10^, ^11^, ^12^ The strongest evidence of efficacy in reducing the prevalence of CAA was found in the **R**andomized controlled trial to **A**ssess **I**mmunoglobulin plus **S**teroid **E**fficacy (RAISE) conducted in Japanese children predicted to be at high risk for IVIG resistance using the Kobayashi score.^9^ The RAISE trial excluded patients with baseline CAA, and safety and efficacy of adjunctive steroids in this population remain.^1^^,^^2^^,^^8^ Randomized trials of cyclosporine in high-risk Japanese children, as well as in trials of infliximab and etanercept in standard-risk North American children, have also shown potential benefit for these adjunctive agents when added to IVIG.^10^, ^11^, ^12^

The Kobayashi score and other Japanese risk scores for IVIG resistance do not perform well for the identification of high-risk populations outside of Japan.^13^, ^14^, ^15^ The strongest predictor of both CAA persistence and adverse cardiac complications in North American children is CAA *z*-score at diagnosis.^14^^,^^16^, ^17^, ^18^, ^19^ Although different adjunctive anti-inflammatory treatments have been proposed for patients with CAA at diagnosis, no high-quality clinical trials have directly compared efficacy adjunctive treatments with one another.^10^^,^^20^, ^21^, ^22^, ^23^ The objective of this cohort study was to determine the association between acute anti-inflammatory treatment regimens and medium-term CA outcomes and risk for MACE in patients with KD with ≥medium CAA at presentation using a pragmatic, multicenter, registry dataset.

## Methods

### Data registry and patient population

In this multicentre, retrospective study, we analyzed data from the International Kawasaki Disease Registry (IKDR) database. The dataset was generated from the collaboration of 34 centres in the United States, Canada, and Taiwan. Medium-term outcomes and patient-level risk factors for CAA regression to normal internal lumen diameter and MACE from the IKDR have recently been reported.^24^ This article focuses on the association between acute adjunctive anti-inflammatory treatments and medium-term outcomes.

The inclusion criteria for this study were ≥medium CAA in any CA segment as defined in the 2017 AHA guidelines (*z*-score ≥5), treatment with IVIG (2 g/kg), at least 1 echocardiogram performed within 3 days of initial IVIG therapy, and a minimum of 1 follow-up echo ≥6 weeks after illness onset. In addition, patients were excluded from the registry if they were seen at the participating institution for a single consultation (or in acute phase only) or were followed up for the first time at the participating centre >3 months after the acute phase. For the purpose of this study, data harvest from the registry was performed on March 1, 2019, and included all follow-up information through December 31, 2018.

### Data collection

Demographic, clinical, and cardiac imaging data were collected by a retrospective review of institutional databases; images were not reviewed. Clinical data included demographics, date of KD onset, duration of fever, treatment centre, KD diagnostic criteria met, initial KD treatments, and convalescent KD therapies over follow-up. First-line treatment at all participating institutions during the study time period was IVIG (2 g/kg) and aspirin.^1^ The indications and timing of adjunctive anti-inflammatory medications varied by centres and era, as all treatment decisions were part of routine clinical care rather than a protocol.^25^^,^^26^ Aspirin, infliximab, and steroid dosage and duration of therapy varied between sites. The registry dataset does not allow for evaluation of the indication for adjunctive therapy (eg, IVIG resistance, CAA at presentation) or the timing of adjunctive therapy in relation to when CAA developed. Anticoagulation with warfarin or low-molecular-weight heparin for patients with giant aneurysms (CA *z*-score ≥10 or absolute dimension >8 mm) is recommended in AHA guidelines but was not followed in all cases.^1^^,^^26^ This study was conducted with the approval from the institutional review board from all participating sites with requirement for consent waived.

### Imaging data

Echocardiographic data were collected from reports produced at the time of the study at each institution. CA measurements for the following segments were evaluated: left main CA, proximal left anterior descending (LAD), proximal right CA (RCA), or circumflex CA (LCX). Using the reported measurements and body surface area, *z*-scores for each segment were calculated using *z*-score equations derived from previously described normative data. We used the 2017 AHA criteria to classify CAA as small (*z*-score = 2.5-4.9), medium (*z*-score = 5 to <10), and large/giant (*z*-score ≥10 or absolute dimension ≥8 mm).^1^ Patients with small CAA at diagnosis were excluded. The maximum *z*-score was defined as the higher value between the RCA and LAD *z*-score on each echocardiogram. Analyses included the CA size and *z*-score at the initial echocardiogram, the maximum RCA or LAD *z*-score over follow-up, and the CA size and *z*-score at the most recent echocardiogram. Patients with maximum *z*-score ≥2.5 in both the RCA and LAD were classified as having bilateral CAA.

### Patient outcomes

The primary outcome measures were time to CAA regression and time to MACE. CAA regression was defined as regression of all CA segments to internal lumen diameter of *z*-score <2.5; this criterion is not meant to imply normalization of the structure of the CA wall. Patients were classified as having MACE if they had any of the following events: myocardial infarction, CA thrombosis, CA bypass graft, percutaneous CA intervention, cardiac death, or orthotopic heart transplant.

### Treatment groups

We categorized treatment groups based on anti-inflammatory treatment given during acute illness as follows: (1) single-dose IVIG only; (2) multiple IVIG doses (≥2 doses); (3) single IVIG + infliximab; (4) single IVIG + corticosteroids; (5) multiple IVIG + corticosteroids; (6) multiple IVIG + infliximab; and (7) multiple IVIG + corticosteroids + infliximab. The corticosteroid dosing categories included both intravenous pulse-dose corticosteroids and lower-dose, longer duration dosing as described by Kobayashi et al. in the RAISE trial.^9^ Data regarding additional adjunctive anti-inflammatory treatment, including use of cyclosporine, cyclophosphamide, and etanercept, were collected and analyzed but not used for patient classification, as these medications were rarely used. Treatments were considered early adjunctive therapy if they were given within 3 days of initial IVIG. Acute anti-inflammatory treatments given >3 days after initial IVIG were considered late/rescue therapy.

### Statistical analysis

Continuous variables were reported using means and standard deviations, or medians and interquartile ranges, as appropriate. Frequencies and proportions were reported for categorical variables. Between-group differences in continuous variables were assessed using Wilcoxon rank-sum tests, and for categorical variables with Fisher’s exact tests.

Time to CAA regression was analyzed using the Kaplan-Meier method, and freedom from MACE using a cumulative incidence function. Multivariable Cox regression models for CAA regression and for MACE were constructed using variables selected a priori based on clinical relevance. Factors associated with the change in CA *z*-score from baseline to maximal *z*-score over follow-up were evaluated using linear multivariable regression. IVIG resistance was not recorded as a variable in the IKDR database. Multivariable analyses used duration of fever in days after initial IVIG as a proxy for IVIG resistance.

All statistical analyses were performed using SAS version 9.4 (SAS Institute, Cary, NC) and Microsoft R open version 3.3.2 (Redmond, WA).

## Results

Of 1652 patients in the IKDR registry, 527 (32%) met study eligibility criteria. The majority of exclusions were due to the maximum CA *z*-score <5 (n = 855) or no echocardiogram within 3 days of initial IVIG (n = 225); other reasons for exclusion were lack of IVIG treatment (n = 30) and missing treatment data (n = 15).

Among the 7 treatment groups, age and sex were similar (Table 1). The median duration of fever before IVIG treatment varied among groups from 6 to 10 (median) days. Duration of fever after IVIG also differed among groups and was longest in the 4 treatment groups that included multiple IVIG infusions. Baseline CA *z*-scores differed among treatment groups and were higher in the corticosteroid groups. At baseline, 199 patients (38%) had large/giant CAA and 328 patients (62%) had medium-sized CAA. Corticosteroids and infliximab were the most used adjunctive therapies. Adjunctive anti-inflammatory medications other than corticosteroids and infliximab were used in 7.5% of patients, most frequently in the multiple IVIG + corticosteroids and multiple IVIG + corticosteroids + infliximab groups.

**Table 1:** Demographic, baseline coronary, and treatment data

	Total (n = 527)	Single IVIG (n = 243)	Single IVIG + infliximab (n = 21)	Single IVIG + steroids (n = 27)	Multiple IVIG n = 100)	Multiple IVIG + infliximab (n = 17)	Multiple IVIG +steroids (n = 75)	Multiple IVIG + steroids + infliximab (n = 44)	*P* value
*Demographics*									
Male, n (%)	368 (70)	169 (70)	18 (86)	19 (70)	72 (72)	11 (65)	52 (69)	27 (61)	0.49
Age (y), median (IQR)	1.3 (0.4-4.0)	1.1 (0.4-4.0)	1.4 (0.5-3.0)	0.8 (0.4-6.8)	1.7 (0.5-3.9)	0.8 (0.3-3.4)	1.1 (0.4-4.1)	1.3 (0.4-5.0)	0.93
Age <6 mo, n (%)	138 (26)	126 (26)	5 (24)	9 (33)	23 (23)	5 (29.4)	22 (29)	11 (25)	0.45
Complete KD criteria, n (%)	265/514 (52)	106/236 (45)	10/19 (53)	9/27 (33)	54/98 (55)	8/17 (47)	51/74 (69)	27/43 (63)	<0.001
Total days of fever, median (IQR)	11 (8-15)	9 (7-13)	9 (6-14)	11 (8-16)	11 (8-15)	12 (9-13)	14 (9-17)	16 (12-21)	<0.001
Days of fever before IVIG, median (IQR)	7 (5-11)	8 (6-12)	8 (5-11)	10 (6-16)	7 (5-10)	7 (5-9)	7 (5-10)	6 (5-10)	<0.001
Days of fever after IVIG, median (IQR)	1 (0-4)	0 (0-1)	1 (0-2)	1 (0-2)	3 (1-5)	3 (1-6)	5 (2-9)	9 (5-14)	<0.001
*Baseline coronary data*									
Baseline RCA *z*-score, mean ± SD	5.4 ± 5.9	5.4 ±4.7	5.9 ±5.7	7.2 ± 9.0	5.1 ±5.1	5.4 ± 4.9	5.9 ± 9.0	4.5 ± 5.2	0.66
Baseline LMCA *z*-score, mean ± SD	3.0 ± 4.1	2.9 ± 2.7	3.0 ± 3.0	5.9 ± 12.8	2.8 ± 2.9	3.2 ± 2.5	2.6 ± 3.6	2.4 ± 3.2	0.01
Baseline LAD *z*-score, mean ± SD	6.2 ± 6.4	6.5 ± 5.4	5.6 ± 6.3	8.8 ± 11.5	5.6 ± 5.9	8.0 ± 9.5	5.6 ± 6.9	4.8 ± 6.2	0.11
Baseline Max *z*-score, mean ± SD	7.4 ± 6.0	7.8 ± 5.2	7.8 ± 6.7	8.2 ± 6.8	7.2 ± 6.0	8.6 ± 9.2	6.5 ± 7.2	5.9 ± 6.2	0.33
Bilateral CAA, n (%)	447 (59)	201 (60)	15 (56)	23 (59)	84 (59)	14 (66)	74 (63)	36 (51)	0.78
*Acute treatments*									
Number of IVIG doses, n (%)									<0.001
1	291 (55)	243 (100)	21 (100)	27 (100)	0 (0)	0 (0)	0 (0)	0 (0)	–
2	202 (38)	0 (0)	0 (0)	0 (0)	91 (91)	16 (94)	64 (85)	31 (71)	–
≥3	34 (6)	0 (0)	0 (0)	0 (0)	9 (9)	1 (6)	11 (15)	13 (30)	–
IV pulse steroids, n (%)	135 (25)	0 (0)	0 (0)	20 (74)	0 (0)	0 (0)	66 (88)	41 (93)	<0.001
Immunotherapy Med∗, n (%)	23/307 (8)	5/131 (4)	0/11 (0)	1/8 (13)	1/11(9)	0/54 (0)	9/53 (17)	7/39 (18)	0.004
Thrombolytic therapy, n (%)	7 (1)	2 (1)	1 (5)	0 (0)	0 (0)	1 (7)	1 (1)	2 (5)	0.19
*Convalescent treatments*									
Lovenox, n (%)	118 (22)	31 (13)	5 (23)	12 (44)	21 (21)	5 (29)	27 (36)	17 (39)	<0.001
Warfarin, n (%)	107/516 (21)	29/239 (12)	2/21 (10)	10 (37)	26/98 (27)	5/16 (31)	17/71 (24)	18/44 (41)	<0.001
Clopidogrel, n (%)	134/515 (26)	36/239 (15)	12/21 (57)	5/27 (19)	28/98 (28.6)	11/16 (69)	20/71 (28)	22/43 (51)	<0.001

CAA, coronary artery aneurysm; IQR, interquartile range; IVIG, intravenous immunoglobulin; KD, Kawasaki disease; LAD, left anterior descending; LMCA, left main coronary artery; Med, medication; RCA, right coronary artery; SD, standard deviation.

∗ Immunotherapy Med = cyclosporine, cyclophosphamide, or etanercept.

An increase in CA *z*-score from baseline to maximal *z*-score over follow-up was evaluated for both early adjunctive and late/rescue therapies (Table 2). Compared with IVIG alone, none of the early adjunctive therapy groups were associated with worsening in *z*-score over follow-up when adjusting for baseline CA size and patient factors (Table 3). Late/rescue therapy, particularly the use of multiple rescue medications, was associated with a greater increase in *z*-score over follow-up compared with IVIG alone. In multivariable regression, independent factors associated with a larger increase in CAA during follow-up included the presence of bilateral CAA, larger CAA size at baseline, and a greater number of days of fever before and after IVIG.

**Table 2:** Coronary and clinical outcomes

	Total (n = 527)	Single IVIG only (n = 243)	Single IVIG + infliximab (n = 21)	Single IVIG + steroids (n = 27)	Multiple IVIG only (n = 100)	Multiple IVIG + infliximab (n = 17)	Multiple IVIG + steroids (n = 75)	Multiple IVIG + steroids + infliximab (n = 44)	*P* value
LAD max *z*-score, mean (SD)	11.5 (10.0)	10.0 (7.7)	9.9 (7.7)	13.9 (14.2)	10.4 (8.0)	15.3(16.6)	13.4 (12.1)	16.3 (13.5)	<0.001
RCA max *z*-score, mean (SD)	9.4 (8.3)	7.6 (5.8)	9.8 (8.3)	12.4 (11.9)	8.5 (6.8)	9.1 (7.4)	12.4 (12.0)	14.2 (9.3)	<0.001
Death, n (%)	2 (0.4)	1 (0.3)	0 (0)	0 (0)	1 (1.0)	0 (0)	0 (0)	0 (0)	−
Heart transplant, n (%)	1 (0.2)	0 (0)	0 (0)	1 (4)	0 (0)	0 (0)	0 (0)	0 (0)	−
CA thrombosis, n (%)	28 (5)	4 (2)	1 (5)	3 (11)	5 (5)	3 (18)	8 (11)	4 (9)	−
CA stenosis, n (%)	31 (6)	12 (5)	0 (0)	5 (19)	4 (4)	1 (6)	4 (5)	5 (11)	−
CABG, n (%)	4 (0.8)	1 (0.4)	0 (0)	0 (0)	0 (0)	1 (6)	2 (2.7)	0 (0)	−
Catheter intervention, n (%)	7 (1)	3 (1)	0 (0)	0 (0)	3 (3)	1(6)	0 (0)	0 (0)	−

CA, coronary artery; CABG, coronary artery bypass graft; IVIG, intravenous immunoglobulin; LAD, left anterior descending; RCA, right coronary artery; SD, standard deviation.

**Table 3:** Factors associated with change in coronary artery *z*-score over follow-up

Variable	Hazard ratio (95% CI)	*P* value
Early∗ treatment (ref: single IVIG only)		
IVIG + steroids (n = 21)	−0.4 (−4.6 to 3.8)	0.84
IVIG + infliximab (n = 27)	2.62 (−4.0 to 9.3)	0.44
Multiple IVIG (n = 132)	−0.8 (−3.5 to 1.8)	0.53
Multiple IVIG + infliximab (n = 9)	1.3 (−5.9 to 8.5)	0.73
Multiple IVIG + steroids (n = 39)	−1.1 (−4.8 to 2.6)	0.57
Multiple IVIG + infliximab + steroids (n = 3)	2.8 (−6.8 to 12.3)	0.93
Late/rescue treatment (ref: single IVIG only)		
IVIG + steroids (n = 21)	4.6 (0.5 to 8.7)	0.03
IVIG + infliximab (n = 27)	−1.6 (−8.0 to 4.9)	0.64
Multiple IVIG (n = 100)	1.2 (−1.5 to 3.9)	0.39
Multiple IVIG + infliximab (n = 17)	3.5 (−2.2 to 9.2)	0.23
Multiple IVIG + steroids (n = 75)	5.4 (2.2 to 8.5)	0.57
Multiple IVIG + infliximab + steroids (n = 44)	6.4 (2.75 to 10.1)	0.55
Age at diagnosis (y)	0.90 (−0.2 to 2.0)	0.93
Maximum CAA *z*-score at first echo (per unit increase)	−0.2 (−0.3 to 0.1)	<0.001
Days of fever before IVIG	0.3 (0.1 to 0.4)	<0.001
Days of fever after IVIG	0.2 (0.1 to 0.4)	0.002
Bilateral CAA at diagnosis (ref: unilateral CAA)	3.4 (1.7 to 5.1)	<0.001

CAA, coronary artery aneurysms; IVIG, intravenous immunoglobulin.

∗ Treatments were considered early adjunctive therapy if they were given within 3 days of initial IVIG.

**Figure 1:**
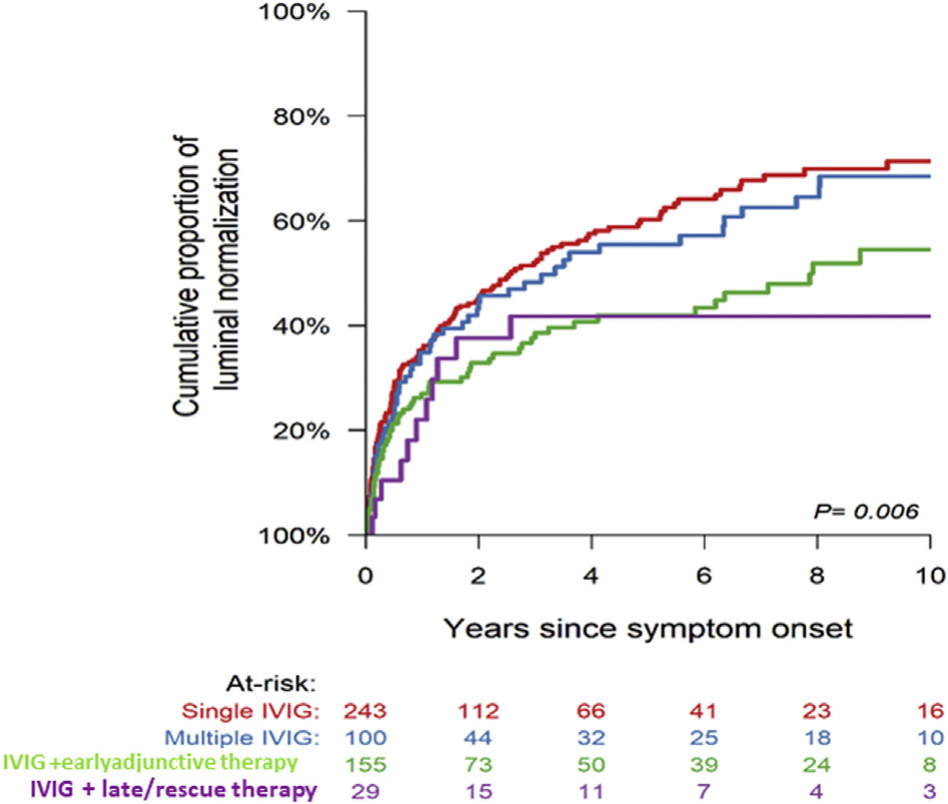
Comparison of the proportion of patients with coronary artery aneurysm regression to normal internal lumen diameter among treatment groups; early adjunctive therapy = treatments given ≤3 days after initial IVIG; late/rescue therapy = treatments given >3 days after initial IVIG. IVIG, intravenous immunoglobulin.

The median duration of follow-up for the entire cohort was 8.9 years. The cumulative incidence of CAA regression to normal internal lumen diameter (CA *z*-score < 2.5 for all CA segments) according to the treatment group is shown in Figure 1. Among all patients, the proportion of patients who have CAA regression to normal internal lumen diameter was 26.5% at 1 year and 46.5% at 5 years. In unadjusted analysis, the single IVIG and multiple IVIG treatment groups had a shorter time to CAA regression compared with groups receiving adjunctive therapy. In Cox multivariable regression analysis (Table 4), when controlling for previously established factors associated with likelihood of CAA regression, none of the early adjunctive therapy groups differed significantly from the IVIG-alone group with respect to time to CAA regression. There was a trend towards shorter time to CAA regression among patients who received early adjunctive therapy with multiple IVIG + steroids compared with IVIG alone (*P* = 0.07). In contrast, the late/rescue therapy groups including multiple IVIG infusions + steroids or multiple IVIG infusions + steroids + infliximab were each associated with longer time to CAA regression. Larger baseline CAA *z*-score, bilateral CAA at diagnosis, and older age at acute KD were independent factors associated with longer time to CAA regression.

**Table 4:** Factors associated with CAA resolution in Cox multivariable regression

Variable	Hazard ratio (95% CI)	*P* value
Early adjunctive treatment (ref: single IVIG only)		
IVIG + steroids (n = 21)	1.3 (0.5-3.2)	0.54
IVIG + infliximab (n = 27)	1.3 (0.4-4.3)	0.60
Multiple IVIG (n = 132)	1.4 (0.8-2.2)	0.23
Multiple IVIG + infliximab (n = 9)	1.5 (0.4-5.8)	0.60
Multiple IVIG + steroids (n = 39)	2.0 (0.9-4.2)	0.07
Late/rescue treatment (ref: single IVIG only)		
IVIG + steroids (n = 21)	0.6 (0.3-1.5)	0.30
IVIG+ infliximab (n = 27)	0.93 (0.3-2.7)	0.88
Multiple IVIG (n = 100)	0.8 (0.5-1.3)	0.44
Multiple IVIG + infliximab (n = 17)	0.3 (0.1-1.1)	0.06
Multiple IVIG + steroids (n = 75)	0.5 (0.3-0.9)	0.02
Multiple IVIG + infliximab + steroids (n = 44)	0.4 (0.2-0.8)	0.01
Age at diagnosis (y)	0.90 (0.86-0.94)	<0.001
Maximum CAA *z*-score at first echo (per unit increase)	0.92 (0.89-0.94)	<0.001
Days of fever before IVIG	0.99 (0.96-1.01)	0.30
Days of fever after IVIG	0.98 (0.95-1.02)	0.31
Bilateral CAA at diagnosis (ref: unilateral CAA)	0.40 (0.89-0.94)	<0.001

CAA, coronary artery aneurysm; CI, confidence interval; IVIG, intravenous immunoglobulin.

**Figure 2:**
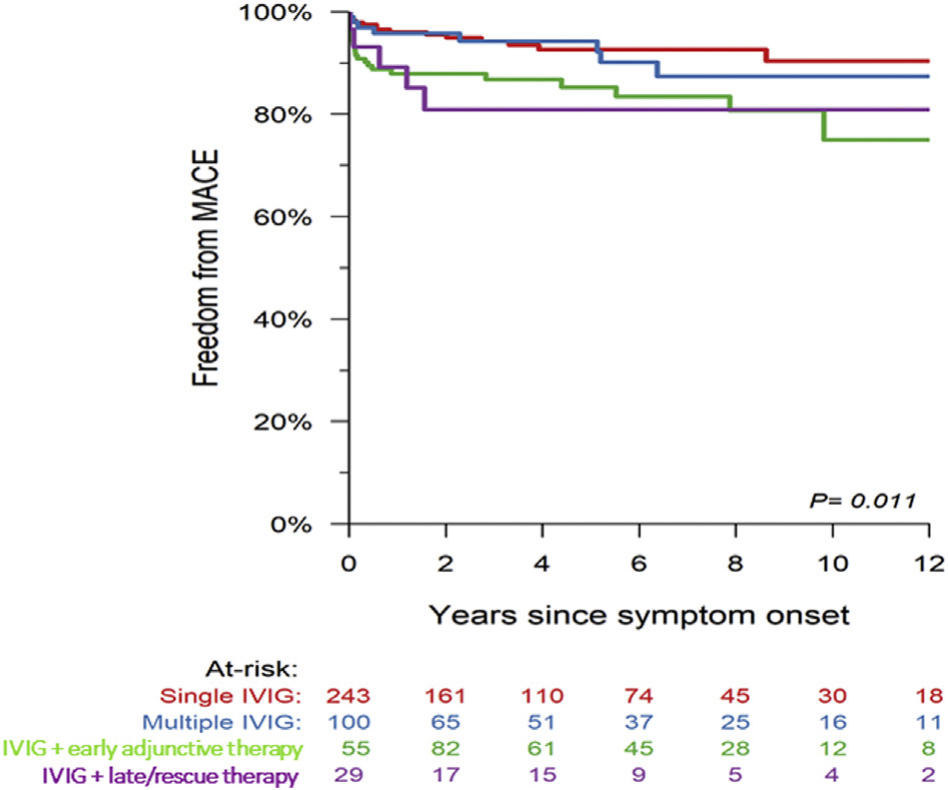
Kaplan-Meier estimation of the proportion of patients with freedom from major adverse cardiac events (MACE) among treatment groups; early adjunctive therapy = treatments given ≤3 days after initial IVIG; late/rescue therapy = treatments given >3 days after initial IVIG. IVIG, intravenous immunoglobulin.

Freedom from MACE based on treatment group is shown in Figure 2. A total of 73 MACEs occurred in 51 patients and included 2 (0.4%) deaths, 1 (0.1%) heart transplant, 4 (1.9%) CA bypass graft, 7 (1.3%) catheter-based CA interventions, 28 (7.3%) CA thromboses, and 31 (7.3%) CA stenoses. In unadjusted analysis, freedom from MACE differed among treatment groups with the following 5-year MACE-free survival rates: single IVG 92.6% (87.6%, 95.7%), multiple IVIG 94.3% (86.6%, 97.6%), IVIG + early adjunctive therapy 85.2% (77.5%, 90.4%), and IVIG + rescue therapy 80.9% (59.8%, 91.6%). In multivariable analysis of MACE (Table 5), a larger CAA *z*-score at diagnosis and higher number of days of fever before and after IVIG were associated with MACE. After controlling for baseline factors, none of the early adjunctive treatment groups were associated with time to MACE. Treatment with multiple IVIG +steroids trended towards a protective effect on MACE but did not reach statistical significance (*P* = 0.07). None of the late/rescue therapy groups were associated with risk of MACE.

**Table 5:** Factors associated with major adverse cardiac events in Cox multivariable regression

Variable	Hazard ratio (CI)	*P* value
Early adjunctive treatment (ref: single IVIG only)		
IVIG + steroids	1.0 (0.3-3.0)	0.93
IVIG + infliximab	3.3 (0.2-49.0)	0.38
Multiple IVIG only	0.5 (0.2-1.5)	0.21
Multiple IVIG + infliximab	3.2 (0.5-20.7)	0.23
Multiple IVIG + steroids	0.2 (0.04-1.2)	0.07
Multiple IVIG + infliximab + steroids	No events	–
Late/rescue treatment (ref: single IVIG only)		
IVIG + steroids	2.9 (0.9-9.5)	0.07
IVIG + infliximab	0.5 (0.02-12.0)	0.68
Multiple IVIG only	1.4 (0.4-4.2)	0.58
Multiple IVIG + infliximab	1.6 (0.2-13.6)	0.66
Multiple IVIG + steroids	2.8 (0.9-8.8)	0.08
Multiple IVIG + infliximab + steroids	2.6 (0.7-9.6)	0.15
Age at diagnosis (y)	1.03 (0.95-1.12)	0.53
Maximum CAA *z*-score at first echo (per unit increase)	1.04 (1.00-1.13)	<0.001
Days of fever before IVIG	1.04 (1.00-1.08)	0.03
Days of fever after IVIG	0.98 (0.95-1.02)	0.05
Bilateral CAA at diagnosis (ref: unilateral CAA)	6.90 (0.93-51.26)	0.06

CAA, coronary artery aneurysm; CI, confidence interval; IVIG, intravenous immunoglobulin.

## Discussion

We evaluated the association of acute anti-inflammatory treatment with medium-term outcomes of patients with KD with ≥medium-sized CAA and found that CAA regression to normal CA lumen diameter and MACE depend largely on patient factors, including later treatment and age, and baseline severity of CA artery enlargement. Medium-term outcomes are less strongly associated with an acute anti-inflammatory treatment regimen after adjusting for patient factors. Indeed, in this study, we found no associations between persistence of CAAs, MACE, or progression in CAA *z*-score and any of the early, adjunctive treatment regimens. However, late/rescue adjunctive therapy, typically given to patients with persistent fever and/or enlarging CAA after initial IVIG treatment, was associated with worse outcomes. Because of confounding by indication, we were unable to draw firm inferences about the comparative efficacy of various early adjunctive anti-inflammatory treatments.

Extensive practice variation in acute anti-inflammatory therapy for patients with KD with medium or larger CAA is present and is attributable to a relative paucity of data from clinical trials and cost-effectiveness studies. Because of lack of evidence-based data in patients with CAA at diagnosis, neither the indications for adjunctive therapy nor the choice of an anti-inflammatory agent is standardized across institutions. The 2017 AHA guidelines suggest that patients deemed high risk for development of CAA may benefit from adjunctive therapy, but do not make further recommendations on a pharmacological agent or specific indications.^1^ Moreover, centres vary in their indications for adjunctive early therapy.^12^^,^^24^^,^^27^ For example, some centres use early adjunctive therapies for high-risk patients based on clinical risk scoring systems, CAA at diagnosis, or other clinical features (presentation with shock, age <6 months, etc.), whereas other centres administer only IVIG as early therapy to all patients, augmenting with additional anti-inflammatory therapy solely on the basis of response to the initial IVIG infusion.^28^ Japanese trials of early adjunctive therapy were designed to exclude patients with CAA at diagnosis, and no randomized controlled trial has compared treatment options for patients with KD who present with CAA.^9^^,^^11^ Thus, there is a critical knowledge gap regarding comparative effectiveness of corticosteroids, infliximab, multiple doses of IVIG, and other adjunctive anti-inflammatory medications in the patients with KD with CAA at diagnosis.

Our study cohort with medium or large/giant CAA had frequent persistence of CAA (approximately 50%) and a high rate of significant burden of MACE occurring in approximately 1 in 10 patients, findings that are concordant with published literature.^4^^,^^5^^,^^29^ Prior Japanese surveys of patients with KD with giant CAA have shown adverse cardiac event rates of 35%-40% at 10 years and 74% at 30 years after acute KD.^4^ As previously described by McCrindle et al.^24^ using the IKDR database and other studies, larger CAA size at diagnosis and bilateral CAA are the strongest factors associated with CAA persistence and MACE. This study is unique in attempting to identify the effect of acute treatment regimens on outcomes in a large cohort of patients with ≥medium CAA. When evaluating early adjunctive therapies (given within 3 days of initial IVIG), we found that CAA regression and MACE were not associated with the specific adjunctive therapies. A randomized controlled trial is necessary to definitively answer the question of efficacy of adjunctive, early therapy in patients with KD with CAA.

This study provides important data on the question of safety of corticosteroids in patients with CAA, an area of long-standing and ongoing controversy. Steroid therapy has been increasingly used in high-risk patients, but there have been anecdotal concerns that adjunctive corticosteroids increase the risk for CAA rupture in the acute phase and may impair vascular wall remodelling and CAA regression.^30^ This study describes the largest experience to date with steroid therapy in patients with KD with ≥medium CAA (n = 138), including 73 with giant CAA. Importantly, in this large cohort of patients with large and giant CAA, there were no instances of CAA rupture. Moreover, in multivariable analysis of time to CAA regression, corticosteroids were not associated with worse outcomes, suggesting that vascular wall remodelling is not impaired because of steroid therapy.

Several important limitations should be noted. Most important among these is confounding by indication. Patients with worse inflammation and longer duration of fever after treatment received a greater number of adjunctive therapies. We attempted to mitigate this confounding by controlling for all factors available in the registry known to be associated with the CA outcome and by distinguishing between therapies given before or after 3 days of illness (early vs late/rescue therapies). Despite this, and within the early adjunctive groups, there is likely residual confounding by indication related to factors for which we could not control. The occurrence of IVIG resistance was not recorded in the registry. Duration of fever after IVIG was used as a surrogate for IVIG resistance. Additional limitations were inherent in this registry design including limitations in granularity of data that can be collected. There was variability in treatment strategies other than acute anti-inflammatory medications, including anticoagulation practices, as well as variability in indications for CA intervention and in intensity of surveillance for MACEs. The multivariable analysis did not consider the potential endogeneity bias incurred by including follow-up treatment groups in the CAA regression and MACE multivariable models. Finally, as a registry study the data are self-reported by the individual centres and CA measurements were not adjudicated in a core laboratory. Timing of follow-up echocardiograms was not consistent between groups.

In conclusion, practice variation is widespread in acute anti-inflammatory therapy for patients with KD with ≥medium CAA in IKDR centres. In the largest experience to date with steroid therapy in patients with KD with ≥medium CAA, we found no cases of CAA rupture and no adverse effects on CAA regression rate, suggesting that steroid therapy is safe in this patient population. Because sicker patients received more adjunctive therapies, our ability to determine the relative efficacy of therapeutic regimens in patients with medium or large/giant CAA was limited but any chosen therapy was associated with a similar rate of progression. In the future, large randomized trials are needed to assess the comparative efficacy of acute adjunctive anti-inflammatory treatment regimens in patients with KD who present with medium to large CAA.

## Appendix 1

List of additional members of the International Kawasaki Disease Registry (IKDR). We acknowledge our IKDR members (in alphabetical order):

Carolyn A. Altman, MD (Texas Children’s Hospital, Baylor College of Medicine, Houston, Texas, USA), Brett R. Anderson, MD, MBA, MS (College of Physicians and Surgeons, Columbia University, New York, USA), Mikayla Beckley, BS (Seattle Children’s Research Institute, Seattle, Washington, USA), Elizabeth Braunlin, MD, PhD (University of Minnesota, Minneapolis, Minnesota, USA), Jane C. Burns, MD (University of California San Diego, Rady Children’s Hospital-San Diego, San Diego, California, USA), Michael R. Carr, MD (Ann and Robert H. Lurie Children’s Hospital of Chicago, Chicago, Illinois, USA), Nadine F. Choueiter, MD (Albert Einstein College of Medicine/Children’s Hospital at Montefiore, New York, New York, USA), Jessica H. Colyer, MD, MBA (Children’s National Hospital, The George Washington University School of Medicine & Health Sciences, Washington, District of Columbia, USA), Frederic Dallaire, MD, PhD (Centre de Recherche du Centre Hospitalier Universitaire de Sherbrooke, Sherbrooke, Québec, Canada), Sarah D. De Ferranti, MD, MPH (Boston Children’s Hospital, Harvard Medical School, Boston, Massachusetts, USA), Laurent Desjardins, MD (Centre Hospitalier Universitaire Ste-Justine, University of Montreal, Montreal, Québec, Canada), Matthew D. Elias, MD (The Children’s Hospital of Philadelphia, Philadelphia, Pennsylvania, USA), Anne Ferris, MBBS (College of Physicians and Surgeons, Columbia University, New York, USA), Michael Gewitz, MD (Maria Fareri Children’s Hospital at Westchester Medical Center Health, New York Medical College, Valhalla, New York, USA), Therese M. Giglia, MD (The Children’s Hospital of Philadelphia, Philadelphia, Pennsylvania, USA), Steven C. Greenway, MD (Alberta Children’s Hospital, Calgary, Canada), Kevin C. Harris, MD, MHSc (Children’s Heart Centre, University of British Columbia, Vancouver, British Columbia, Canada), Kevin D. Hill, MD, MSc (Duke University Medical Center, Durham, NC, USA), Michelle Hite (Children’s Hospital Colorado, University of Colorado School of Medicine, Aurora, USA), Thomas R. Kimball, MD (Heart Institute, Cincinnati Children’s Hospital Medical Center, Cincinnati, Ohio, USA), Shelby Kutty, MD (Children’s Hospital & Medical Center of Omaha, Omaha, Nebraska, USA), Lillian Lai, MD, MHA (Children’s Hospital of Eastern Ontario, Ottawa, Ontario, Canada), Simon Lee, MD (The Heart Center at Nationwide Children's Hospital, Columbus, Ohio, USA), Ming-Tai Lin, MD, PhD (National Taiwan University, Taipei, Taiwan), Tisiana Low, MD, MSc (University of Toronto, The Hospital for Sick Children, Toronto, Ontario, Canada), Andrew S. Mackie, MD, MSc (Stollery Children’s Hospital, Edmonton, Alberta, Canada), Wadi Mawad, MD (Montreal Children’s Hospital, McGill University, Montreal, Québec, Canada), Mathew, MSc (University of Toronto, The Hospital for Sick Children, Toronto, Ontario, Canada), Kimberly E. McHugh, MD (Medical University of South Carolina, Charleston, South Carolina, USA), Tapas Mondal, MD (McMaster Children’s Hospital, Hamilton, Ontario, Canada), Kimberly Myers, MD (Alberta Children’s Hospital, Calgary, Canada), Michael A. Portman, MD (Seattle Children’s Research Institute, Seattle, Washington, USA), Claudia Renaud, MD (Montreal Children’s Hospital, McGill University, Montreal, Québec, Canada), Rosie Scuccimarri, MD (Montreal Children’s Hospital, McGill University, Montreal, Québec, Canada), S. Kristen Sexson Tejitel, MD, PhD, MPH (Texas Children’s Hospital, Baylor College of Medicine, Houston, Texas, USA), Karen M. Texter, MD (The Heart Center at Nationwide Children’s Hospital, Columbus, Ohio, USA), Deepika Thacker, MD (Nemours/Alfred I. duPont Hospital For Children, New Castle County, Delaware, USA), Sharon Wagner-Lees, RN-BC, BSN (Children’s Hospital of Los Angeles, California, USA), Kenny Wong, MD (IWK Health Centre, Halifax, Nova Scotia, Canada), Mei-Hwan Wu, MD, PhD (National Taiwan University, Taipei, Taiwan), Varsha Zadokar, MBBS (Nemours/Alfred I. duPont Hospital For Children, New Castle County, Delaware, USA).
